# Ocean acidification influences plant-animal interactions: The effect of *Cocconeis scutellum parva* on the sex reversal of *Hippolyte inermis*

**DOI:** 10.1371/journal.pone.0218238

**Published:** 2019-06-26

**Authors:** Mirko Mutalipassi, Valerio Mazzella, Valerio Zupo

**Affiliations:** Integrated Ecology Department, Benthic Ecology Centre, Stazione Zoologica Anton Dohrn, Ischia, Italy; Universidade dos Acores, PORTUGAL

## Abstract

Ocean acidification (O.A.) influences the ecology of oceans and it may impact plant-animal interactions at various levels. Seagrass meadows located at acidified vents in the Bay of Naples (Italy) are considered an open window to forecast the effects of global-changes on aquatic communities. Epiphytic diatoms of the genus *Cocconeis* are abundant in seagrass meadows, including acidified environments, where they play key ecological roles. A still-unknown apoptogenic compound produced by *Cocconeis* triggers the suicide of the androgenic gland of *Hippolyte inermis* Leach 1816, a protandric hermaphroditic shrimp distributed in *P*. *oceanica* meadows located both at normal pH and in acidified vents. Feeding on *Cocconeis sp*. was proven important for the stability of the shrimp’s natural populations. Since O.A. affects the physiology of diatoms, we investigated if, in future scenarios of O.A., *Cocconeis scutellum parva* will still produce an effect on shrimp’s physiology. Cell densities of *Cocconeis scutellum parva* cultivated in custom-designed photobioreactors at two pH conditions (pH 7.7 and 8.2) were compared. In addition, we determined the effects of the ingestion of diatoms on the process of sex reversal of *H*. *inermis* and we calculated the % female on the total of mature individuals^-1^ (F/mat). We observed significant differences in cell densities of *C*. *scutellum parva* at the two pH conditions_._ In fact, the highest cell densities (148,808 ±13,935 cells. mm^-2^) was obtained at day 13 (pH 7.7) and it is higher than the highest cell densities (38,066 (±4,166) cells. mm^-2^, day 13) produced at pH 8.2. Diatoms cultured at acidified conditions changed their metabolism. In fact, diatoms grown in acidified conditions produced in *H*. *inermis* a proportion of females (F/mat 36.3 ±5.9%) significantly lower than diatoms produced at normal pH (68.5 ±2.8), and it was not significantly different from that elicited by negative controls (31.7 ±5.6%).

## Introduction

*Hippolyte inermis* Leach is a shrimp mainly inhabiting meadows of *Posidonia oceanica* [1] and in other seagrasses [2]. *Hippolyte inermis* is a key component of their food webs, as a link between primary producers, fishes and other carnivores [3]. The shrimp naturally undergoes a process of protandric sex reversal [4,5].

However, it was demonstrated the presence of two distinct reproductive periods, in spring and in fall. The offspring born during the fall period are characterised by males that undergo sex reversal after the next spring recruitment period, producing “alpha” females [6]. On the contrary, the spring period is characterised by offspring consisting of directly developing males and females. The presence of spring early-developed females (“beta” females) contributes to the fall reproductive burst and these “beta” females exhibits the maximum abundance in association with blooms of epiphytic diatoms [7]. The early-developed females demonstrated a peculiar dietetic pattern: in spring, gut contents of “beta” females are dominated by benthic diatoms, among which *Cocconeis* spp. are particularly abundant. In contrast, males and “alpha” females are generalist grazers feeding on common epiphytes of *P*. *oceanica* leaves such as micro and macroalgae, bryozoans and foraminiferids [3]. It has been demonstrated that a) the ingestion of *Cocconeis* diatoms influences in a narrow time window the physiology of *H*. *inermis* [8]; b) the production of “beta females” is triggered by a still unknown lipophilic compound produced by diatoms after wounding and c) the compound has a highly selective apoptogenic power targeted on the shrimp’s androgenic glands (A.G.) [9]. The destruction of the A.G. in males of *H*. *inermis* by apoptosis represents a stabilizing factor for natural populations [7] by triggering an increase of ovigerous females during the fall reproductive season.

Various diatoms dramatically change their productivity and growth dynamics, as well as the composition and concentration of secondary metabolites, in different culture conditions, influenced by light irradiance [10,11], presence of pollutants [12], nutrient limitations [13] and light spectrum [14,15].

Ocean acidification affects the ecology of oceans and the physiology of marine organisms and various direct and indirect effects on the marine biota are forecasted. Concentration of CO_2_ in oceans increased in the last decades due to anthropogenic emissions [16] and a decrease in 0.43 pH units is forecasted to occur over the next century [17]. Thus, the chemistry of oceans and consequently various biological processes will be deeply affected by increasing concentrations of atmospheric carbon dioxide. O.A. combined with such factors as eutrophication and temperature rising, may cause a significant decrease in the abundance and diversity of calcareous algae [18]. Indeed, O.A. may have a deep effects on plant-animal interactions, algal growth, calcification rates of various algae [19,20] as well as other physiological processes such as nutrient uptake [18], and metabolisms [21–24]. In addition, algae living in acidified environments may modify their patterns of production of secondary metabolites which, in turn, may impact marine food webs. In the “Castello” vents off the island of Ischia (Bay of Naples, Italy), considered as a natural laboratory to simulate future O.A. scenarios, previous researchers identified more than 22 diatom genera in the epiphytic community on *P*. *oceanica* leaves among which *Cocconeis* spp. were numerically the dominant ones [25].

## Aims

This study aimed at investigating how O.A. can affect the cell density of *Cocconeis scutellum parva* benthic diatoms simulating the present status (pH 8.2) and a hypothetical future condition (pH 7.7). Since the shrimp *H*. *inermis* is the only known “biological sensor” that is able to track the presence of the active apoptogenic compound produced by the diatom, the effects of *Cocconeis* spp. cultivated at the two pH scenarios on shrimps’ post-larvae diet was examined to test its potential effect on plant-animal interactions. To compare the effects of *C*. *scutellum parva*, *C*. *scutellum posidoniae*, a strictly related variety that has been proved to be able to produce apoptogenic metabolite [26], was also tested as positive control.

## Materials and methods

### Stock cultures and inoculation process

Two *Cocconeis spp*. were taken into consideration, *i*.*e*., *C*. *scutellum parva and C*. *scutellum posidoniae*. All the diatoms used in this investigation belong to our stock culture that were collected in 2014 in Lacco Ameno, Ischia, Italy. Each species was cultured in continuous axenic conditions in 6 mL multi-wells containing 4 mL of Guillard’s *f*/2 medium with silica (Sigma-Aldrich, Milan, Italy). Cultures were kept under controlled conditions in a thermostatic chamber at 18°C with 12:12 light:dark photoperiod. Light was provided by Sylvania GroLux (Osram Sylvania Inc., USA) at an irradiance of 140 μE. m^-2^. s^-1^.

At the 16^th^ day of grow-out, the surface of the multi-wells was almost completely covered by diatoms and cells were scraped and collected by a Pasteur pipette, then pooled in a sterilized beaker; the suspension was divided and transferred into 10 Petri dishes (diameter 7 cm) filled with 50 mL of *f*/2 medium. At the end of the next grow-out phase (16 days), diatoms were collected by gently scraping off (with the aid of a Pasteur pipette) the bottom of the culture vessels. Diatoms were pooled again in a sterilized beaker and the suspension was partitioned into three photobioreactors filled with 2 litres of *f*/2 medium each. Given the adhesive properties of *Cocconeis* spp., only part of diatoms inoculated in each photobioreactor survived each grow-out phase; for this reason, the diatom concentration in the suspension was not determined at the moment of the inoculation [15].

### Seawater carbonate chemistry

Samples of culture medium (50 mL of volume) were collected from each replicate each 3 days to perform analysis of the seawater carbonate chemistry. Salinity was measured with a HI-96822 refractometer for seawater (Hanna Instruments, Woonsocket, Rhode Island, United States). Total alkalinity (TA) and pH (NBS scale; pH_NBS_) was determined using the total Alkalinity mini titrator for water analysis HI-84531-02. Three points electrode calibration (pH 4.01, 7.01 and 8.30 at 25°C) and pump calibration (using a HI 84531–55 calibration standard) were performed before each set of titrations to assure high accuracy. Working temperature (18°C) was automatically adjusted by the instrument using the automatic temperature compensation feature. Samples were analysed immediately after obtained from replicate photobioreactors carefully avoiding the formation of any air bubble in the instrument that could alter the measurement.

Seawater partial pressure of CO_2_ analyses were performed using the CO_2_Sys EXCEL Macro [27–29] from pH_NBS_, TA, temperature, and salinity data. Carbonic acid dissociation constants (i.e., pK_1_ and pK_2_, [30]), ion HSO_4_ constant [31] and borate dissociation constant [32] were used for the computation.

### Photobioreactors

**Fig 1:**
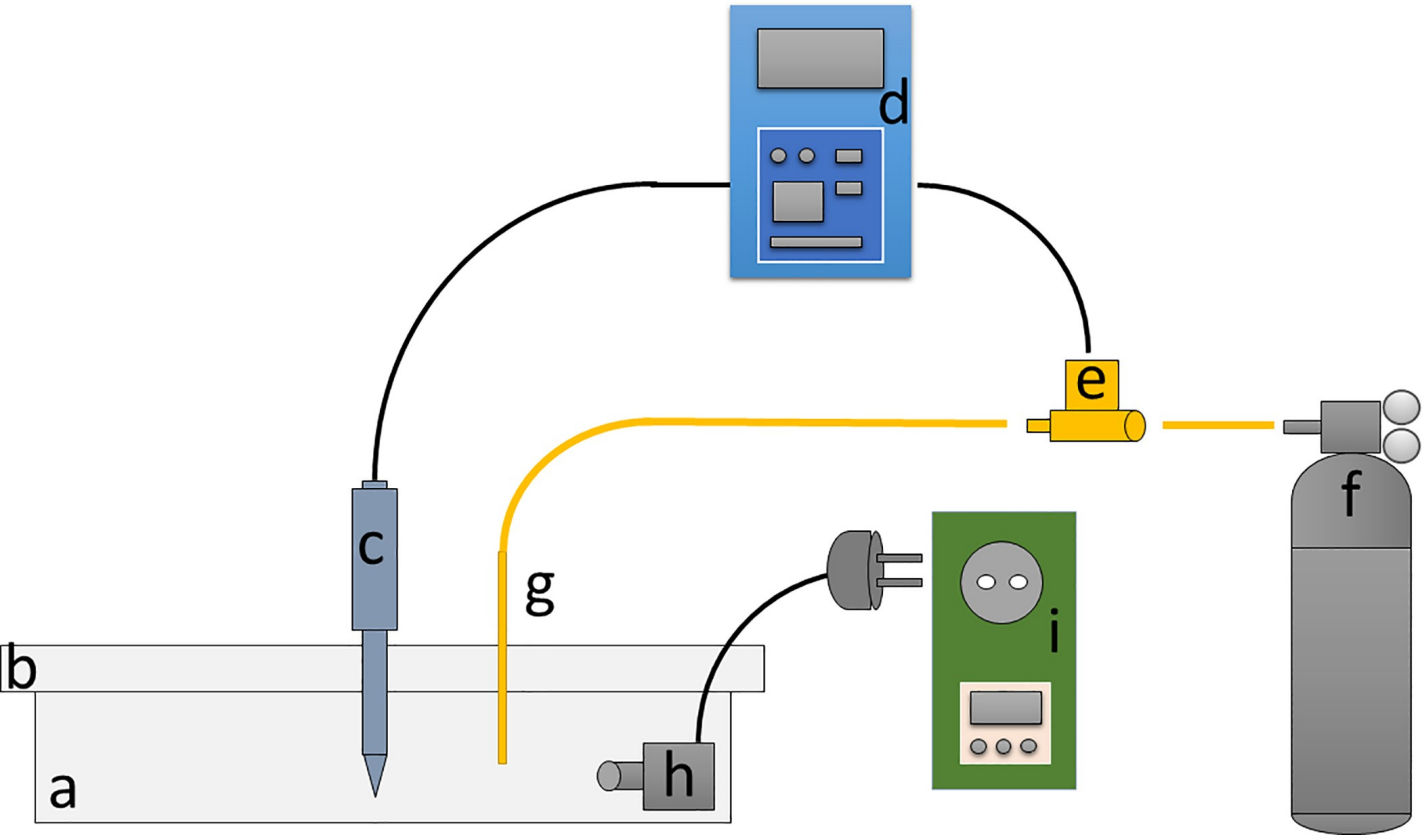
Photobioreactors devised to culture benthic algae at normal and acidified pH. Each reactor was constructed using a Pyrex dish (a) covered with a heat resistant glass plate (b). In the opening at the center of the cover was housed a pH probe (InLab Micro pH, Mettler Toledo; c). The pH probe was connected to a pH controller (pH 201, Aqualight; d) that controls an electronic valve (e). The electronic valve is connected, on one side, to the CO_2_ regulator (CO_2_ Energy, Ferplast; f), and on the other side to a plastic stripette (g), fixed in a secondary opening in the glass cover. A centrifuge pump (Askoll Pure pump 300; h) was placed on one side of the photobioreactor to avoid water stratification and was temporized by a microcontroller (EnerGenie EG-PMS2-LAN; i).

Special photobioreactors adapted for benthic diatoms were *ad-hoc* designed to perform at normal and acidified conditions. Each photobioreactor was assembled using a Pyrex dish with a total volume of 2.4 L (300 mm x 200 mm x 40 mm; Fig 1).

The vessel was covered with a heat resistant glass plate, provided with a narrow opening at its centre, where a pH probe was housed (InLab Micro pH, Mettler Toledo). A secondary opening was placed sideways, where a plastic stripette was fixed. The InLab Micro probe is designed to work even in a reduced volume of water and up to a thickness of 3 mm. A pH controller (pH 201, Aqualight) was connected to the Inlab Micro Probe (via a BNC cable) by an electronic valve which was connected to a CO_2_ regulator (CO_2_ Energy, Ferplast). To avoid water stratification and the formation of any pH gradient along the photobioreactor, a centrifuge pump (Askoll Pure pump 300) was added. The centrifuge pump was temporized by a micro-controller (EnerGenie EG-PMS2-LAN) that cyclically activated the pump each 30 minutes (1 minute on, 29 minutes off). The pH, in both treatments (pH 8.2 and pH 7.7) was regulated by a pH controller that opened and closed the electronic valve, when necessary, dispensing the CO_2_ through a plastic stripette into the photobioreactors. The pH of the medium was checked five times a day to guarantee that pH oscillations were lower than 0.05.

Photobioreactors were used to culture *C*. *scutellum parva* at pH 7.7 and 8.2. In addition, *C*. *scutellum posidoniae* was cultivated at normal pH (8.2). Three replicates were produced for each diatom and condition. The growth of diatoms into photobioreactors continued for 16 days. To estimate the number of cells. mm^-2^ into photobioreactors, 24 microscopy cover-slides (1 cm^2^), glued to a nylon wire in order to facilitate the picking operations, were placed into each photobioreactor. In each cover-slide, 24 areas of 0.04 mm^2^ were randomly selected and examined under the inverted microscope to record the number of cells grown. Every 3 days, 4 cover-slides per replica were collected and examined, and the average number of cells present per surface area was computed. Examined cover slides were trashed to avoid contamination in the culture photobioreactors. Average number of cells for each species of diatoms and standard deviations among replicates were also computed. Diatom transfers and collections were performed under a laminar flow hood and all dishes and culture instruments were previously sterilized at 120°C. All the intact cells of benthic diatoms strongly adhere to the bottom of glass cups 24 hours after the inoculation.

After 16 days the medium in each photobioreactor was removed and the vessels were quickly rinsed with distilled water to remove residual salts. Emptied vessels containing a diatom film on their bottom were immediately frozen at -20°C, then freeze-dried. Dry diatoms were scraped off using an iron blade, then weighed and kept in dry vessels at −20°C up to their use for bioassays on shrimps.

### Bioassays on *Hippolyte inermis*

We followed the techniques described by Zupo and Messina [33] to tests the effects of diatoms on the target shrimp *Hippolyte inermis*. Ovigerous females of *H*. *inermis* were collected in a *Posidonia oceanica* meadow off Castello Aragonese (Island of Ischia, bay of Naples, Italy), sorted on boat and kept in plastic bags to be transferred to the laboratory. Ovigerous females were then transferred into 2 L conical flasks (2 ind. in each flask), containing 1.8 L of filtered seawater and small portions of *P*. *oceanica* leaves which were added to provide a shelter for shrimps. They were kept at 18°C in a thermostatic chamber at a mean irradiance of 250 μmol m^−2^ s^-1^ with a photoperiod of 10:14 h light: dark. Once a sufficient number of larvae has been released by females and collected, the breeders have been returned to the sea.

Larvae produced were collected daily and transferred to 10 conical flasks containing 800 mL (total volume 1 litre) of filtered seawater (pH 8.2) in pools of 80 individuals. Larvae were fed with *Artemia salina* nauplii and *Brachionus plicatilis* (4 individuals per mL) for 7 days. *Artemia salina* nauplii were enriched daily with Algamac Biomarine (Hawthorne, CA, USA). Survival rates were recorded daily, by collecting larvae using a Pasteur pipette and transferring them into a fresh culture medium (filtered sea-water). Larvae already metamorphosed into post-larvae (in about 26 days) were randomly pooled and further divided in groups of 5 replicates of 25 post-larvae for each treatment. Post-larval culture vessels consisted of 14 cm diameters crystallizing dishes containing 400 mL of filtered seawater. Negative controls were fed with a base food composed of equal proportions of SHG “Artemia Enriched”, SHG “Microperle” and SHG “Pure Spirulina” (produced by Super High Group, Ovada, Italy). Treatments were fed with dried cells of *C*. *scutellum parva* cultured at pH 7.7 or 8.2 added to the basic food in a ratio of 2:1 (w/w), according to treatments. Positive control replicates were fed with dried cells of *C*. *scutellum posidoniae* in addition of the base food with the same proportions used for the bioassays. Dry feeds were prepared and stored at -20°C. Each post-larval replicate received daily a 5 mg ration of feed. Post-larvae medium (filtered sea-water) was daily replaced, the crystallizing dishes were washed and shrimps transferred. Post-larvae aged 40 days were sacrificed and fixed in 70% ethanol. Their total body length was measured using millimetric paper under a dissecting macroscope (Leica Z16 APO) and pleopods II were cut, mounted on a slide and observed under an optical microscopy (Leica DMLB) to determine their sex based on the presence/absence of a masculine appendix [34].

### Statistical analyses

Average survival rates in larval cultures were evaluated and plotted by Prism 7 (Graph-Pad Software, La Jolla, USA). Diatom growth curves were computed according to the following equation:

$$Y=(Y_{0}-a).e^{\left(-b.X\right)}+a$$

where:

“a” is the Y value at infinite times;

“Y_0_” is Y value when X is zero;

“b” is the rate constant, expressed in reciprocal of the X axis time units;

Cell densities obtained for *C*. *scutellum parva* at the two pH conditions were compared by a paired t-test. Cell densities and pCO_2_, at each replicate and time interval, were statistically compared in the two pH conditions. The time evolution of cell densities according to the pCO_2_ concentration was evaluated according to the equation:

$$Normalized\;cell\;densities=(Number\;of\;cells.{mm}^{-2}).{p{CO}_{2}}^{-1}$$

in order to relate the actual densities of diatoms to the abundance of carbon. The differences in the time evolution of cell densities in various pH conditions were analysed by a Spearman correlation analysis (Prism 7, Graph-Pad Software, La Jolla, USA).

The percentage of females normalized to the total number of mature individuals (% Female. mature individuals^-1^) was computed. The % female. mature individuals^-1^ (F/mat) index permits to determine the effects of diatom on mature *H*. *inermis*, avoiding the bias due to immature individuals or shrimp with corroded pleopods due to bacterial infections.

The significance of differences among treatments and controls was tested by one-way analysis of variance (ANOVA), adopting the Tukey’s multiple comparisons post-hoc test (Prism software) to the observed F/mat scores.

## Results

### Photobioreactors and diatom cultures

The photobioreactors here developed permitted to keep the pH of the medium constant during the whole experimental period, by adjusting the pCO_2_ with small additions of gas immediately dissolved by the movements of the applied pumps (Table 1).

**Table 1:** Seawater carbonate chemistry variables (mean values ± standard deviation) in photobioreactors at the two pH conditions (pH 7.7 and 8.2).

Conditions	Day	pH_NBS_		TA		pCO_2_	
		Mean	SD	Mean	SD	Mean	SD
pH 8.2	1	7.702	±0.025	3,030.3	±5.5	1,812.2	±76.4
	4	7.701	±0.026	3,025.3	±6.4	1,814.4	±111.4
	7	7.691	±0.044	3,023.7	±12.1	1,837.8	±136.3
	10	7.683	±0.042	3,031.3	±4.6	1,862.1	±168.9
	13	7.703	±0.042	3,023.7	±6.7	1,815.2	±147.5
	16	7.691	±0.046	3,031.3	±6.4	1,843.7	±147.9
pH 7.7	1	8.175	±0.054	3,025.7	±8.1	530.2	±65.7
	4	8.209	±0.055	3,032.3	±7.2	491.1	±65.1
	7	8.208	±0.049	3,032.3	±3.8	488.3	±57.4
	10	8.196	±0.045	3,028.7	±5.0	501.6	±63.8
	13	8.175	±0.050	3,026.3	±7.5	520.9	±68.6
	16	8.177	±0.056	3,026.7	±9.6	524	±62.8

pH_NBS_ = measured pH (NBS scale); TA = total alkalinity; pCO_2_ = CO_2_ partial pressure.

**Fig 2:**
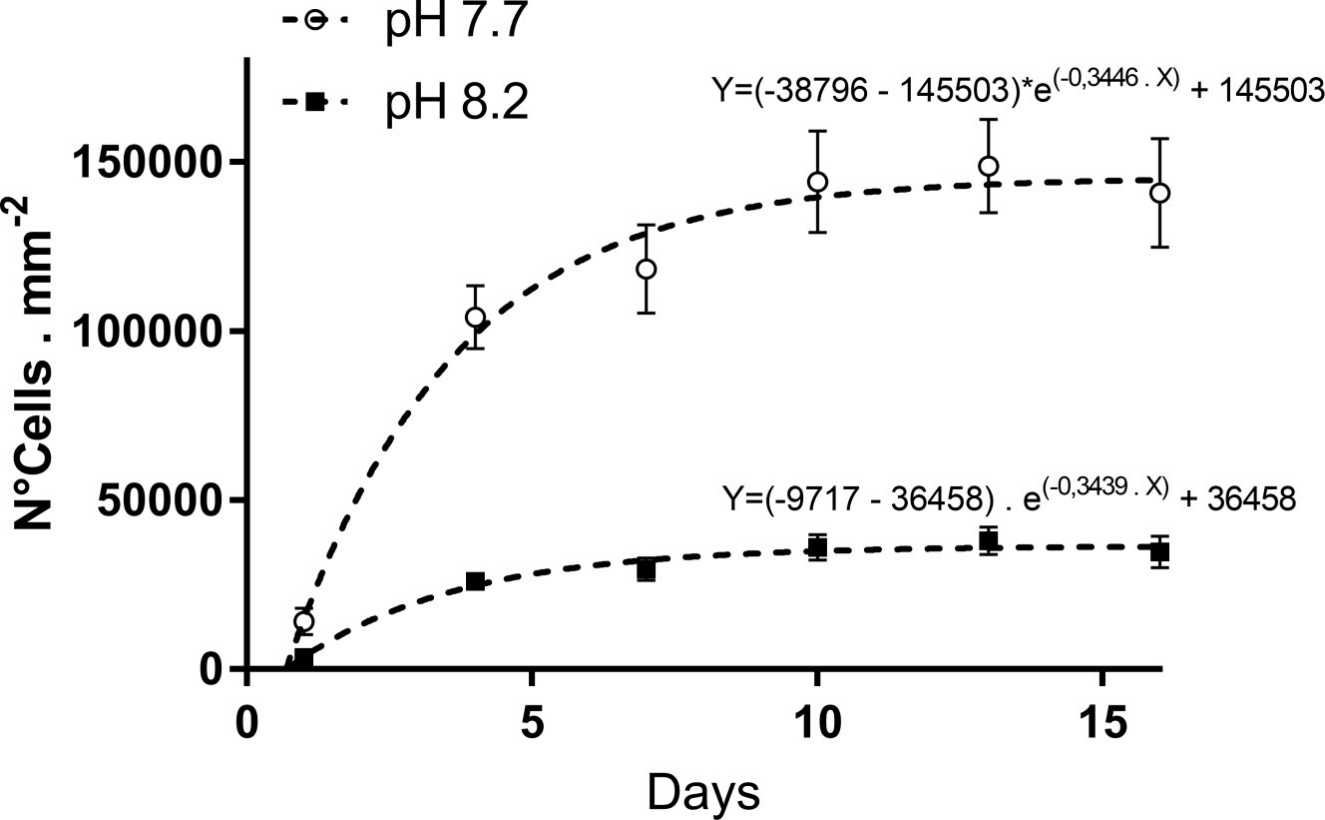
Growth curve of *Cocconeis scutellum parva* at pH 7.7 (white dots) and at pH 8.2 (black square) obtained over 16 days of culture. Error bars indicate the standard deviation among cell counts obtained in the same day in all replicates.

The maximum deviation of the pH from the values set on the control instrument was 0.05. At the end of the culture periods, we observed an average pH of 7.695 (±0.033), 8.19 (± 0.046) and 8.2 (±0.030) in *C*. *scutellum parva* cultures at pH 7.7, at pH 8.2 and in *C*. *scutellum posidoniae* cultures, respectively. The cell density at the beginning of the experiment (24 hours after inoculation) in replicates at pH 7.7 and 8.2 was 14,183 (±3,975) and 3,546 (±994) cells. mm^-2^, respectively (Fig 2).

However, steady state was consistently reached within 10 days of incubation at both tested conditions. *Cocconeis scutellum parva*, cultured at two pH conditions (pH 7.7 and 8.2) produced significant differences in the cell density (t test; P ≤ 0.01). The highest cell densities were recorded at pH 7.7, with 144,283 (±15,048) and 148,808 (±13,935) cells. mm^-2^ reached at the days 10 and 13, respectively. *Cocconeis scutellum parva* cultured at pH 8.2 produced, over the same period of time, cell densities of 36,070 (±3,762) and 38,066 (±4,166) cells. mm^-2^, respectively. The pCO_2_ significantly influenced the cell densities reached in each plate (Spearman test, P ≤ 0.1, rs = 0.9429).

### Bioassay

**Fig 3:**
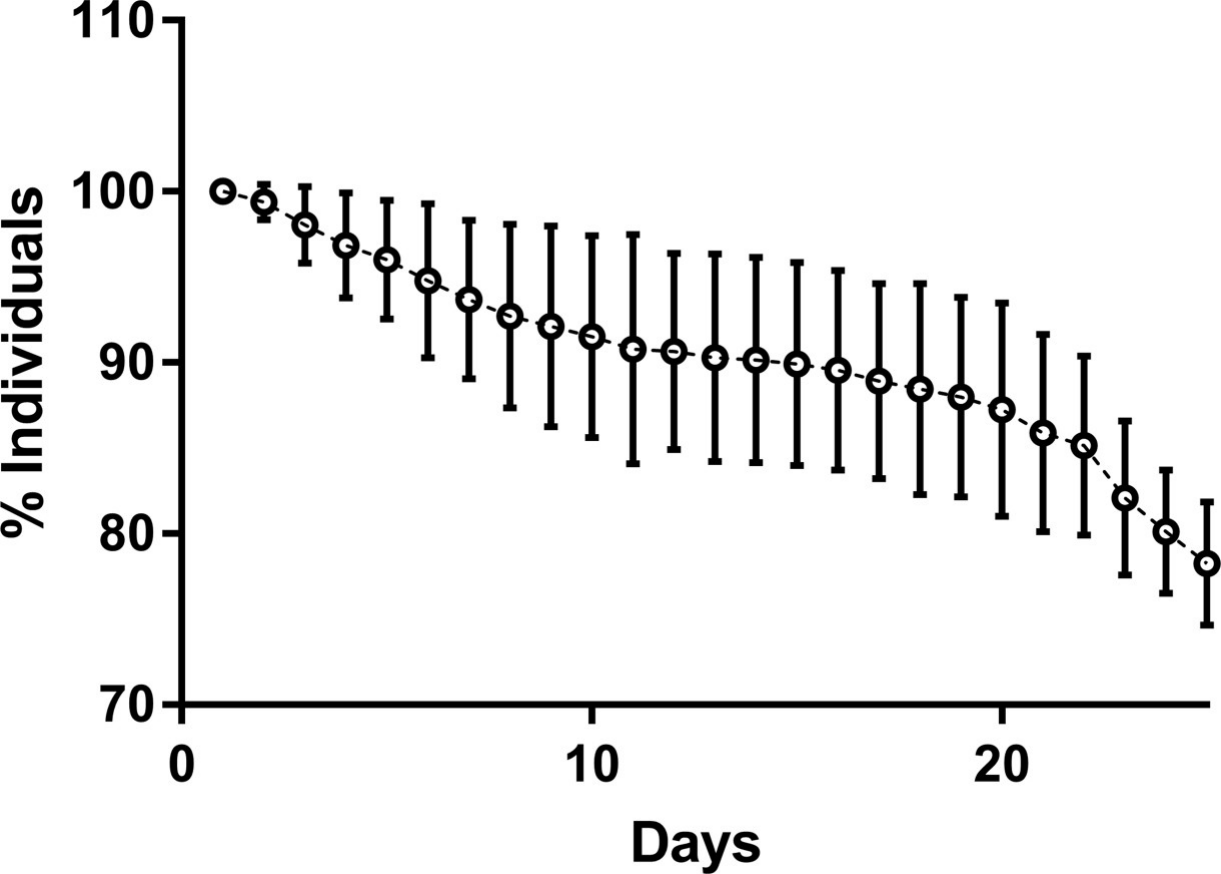
Percent survival rates of *Hippolyte inermis* cultured 26 days in conical flask (10 replicates). Dots indicate the average values and vertical bars their standard deviations.

On average 81.8 (±19.3) larvae were produced by each of ten ovigerous females. During 24.5 (±1.08) days of larval growth, survival rate was 78.26% (±3.6; Fig 3).

After 25 days of larval culture all the individuals settled and metamorphosed into post-larvae.

A survival rate of 80 (±4.9) %, a percentage of 6.4 (±4.6) % immature individuals and an average size of 7.63 (±0.65) mm were recorded in fixed shrimps, at the end of post-larval culture. Treatments fed on *C*. *scutellum parva* cultured in acidified conditions (pH 7.7) produced a survival rate of 87.2 (±7.7) %, a percentage of immatures of 7.2 (±5.9) % and an average size of 7.52 (±0.63) mm. In treatments fed on *C*. *scutellum parva* cultured at normal conditions (pH 8.2) a survival rate of 82.4 (±2.2) %, 4.0 (±4.0) % of immatures and an average size of 7.80 (±0.60) mm were recorded.

Treatments fed on *Cocconeis scutellum posidoniae* produced a survival rate of 86.4 (±5.4) %, a percentage of immature of 4.8 (±1.8) % and an average size of 7.75 (±0.63) mm. During the post-larvae phase, all individuals experiences the same culture conditions and they were cultured contemporaneously in the same thermostatic chamber.

**Fig 4:**
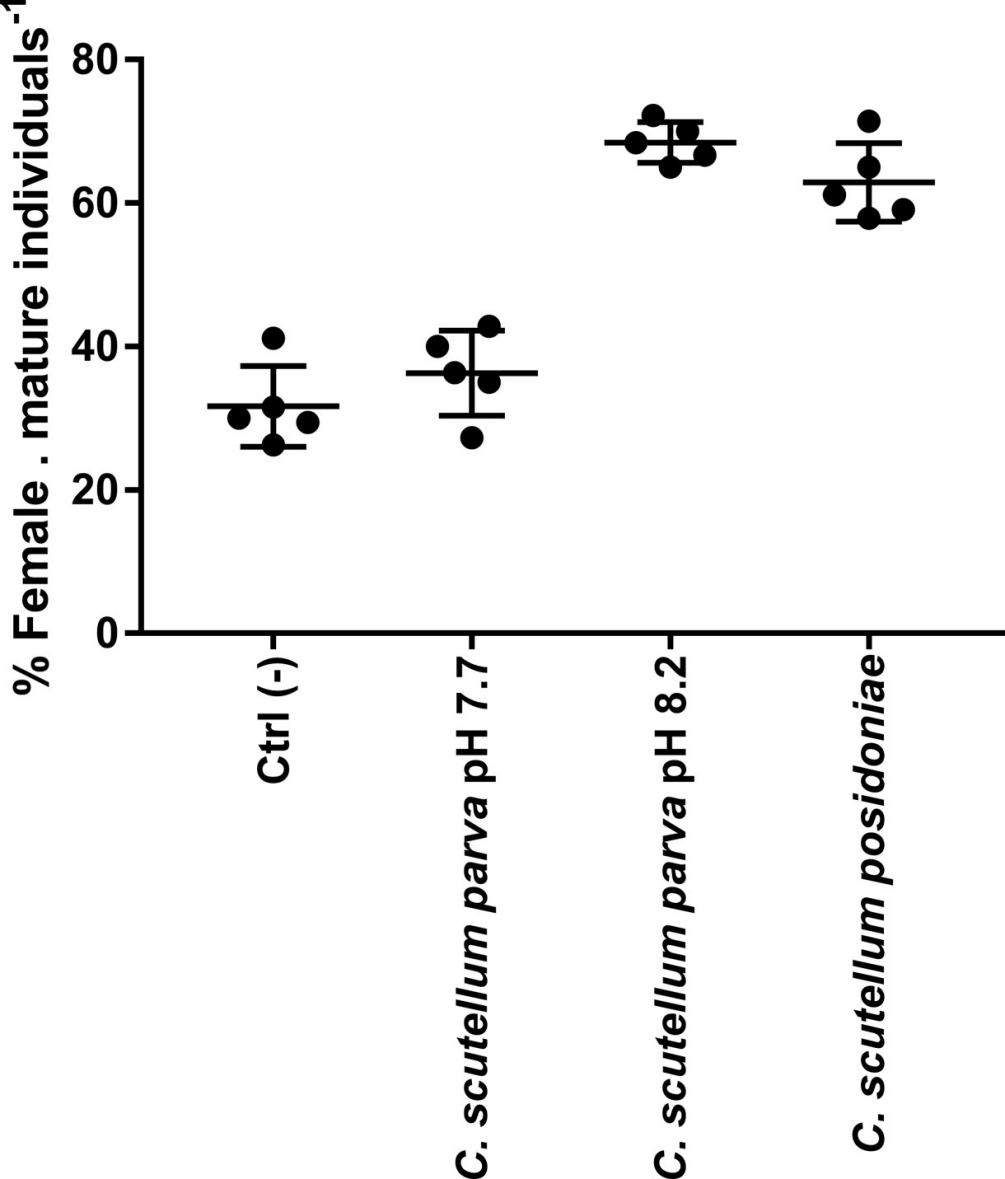
Female/Mature ratio obtained for each diet. Average values, standard deviations and the value of each replicate are reported.

The highest activity (highest ratio of sex reversed individuals, evaluated according to the presence/absence of *masculinae* appendices on shrimp’s pleopods) was measured in replicates fed on *C*. *scutellum parva* cultured at normal conditions (pH 8.2), where a percentage of 68.5 (±2.8) % female. mature individuals^-1^ was recorded (Fig 4). Positive controls produced a high number of females with 63.4 F/mat ±2.8% in replicates fed on *C*. *scutellum posidoniae*. Replicates fed on *C*. *scutellum parva* cultured in acidified conditions (pH 7.7) as well as negative controls, produced a low number of females (36.3 F/mat ±5.9% and 31.7 F/mat ±5.6% respectively) and significant differences among treatments were indicated by ANOVA (P ≤ 0.0001; S1A Table). Notably, no differences in the percentage of F/mat were observed between negative controls and *C*. *scutellum parva* cultured at acidified conditions (Tukey’s, P ≥ 0.05; S1B Table) as well as between *C*. *scutellum parva* cultured at normal conditions and *C*. *scutellum posidoniae* (Tukey’s, P ≥ 0.05; S1B Table). In contrast, significant differences were found between *C*. *scutellum parva* cultured both at normal and acidified conditions and negative controls (Tukey’s, P ≤ 0.0001; S1B Table).

## Discussion

Our results demonstrated that *Cocconeis scutellum parva* cultured at acidified conditions (pH 7.7) produced four times more cells than the same diatoms cultured at normal conditions (pH 8.2). The pCO_2_ recorded at pH 7.7 is quite higher (about 4 times) then the one recorded at pH 8.2 and, similarly, the cell densities recorded at pH 7.7 are significantly higher (about 4 times) than the ones recorded at pH 8.2. In fact, the time trends of cell densities are significantly correlated to the pCO_2_. Our results on the growth of *C*. *scutellum parva* in photobioreactors are in accordance with studies performed at Castello Aragonese meadows, where species populating the vent include a suite of organisms resilient to naturally high concentrations of pCO_2_ and the massive presence of *Cocconeis sp*. may indicate that these species may have a competitive advantage under low pH conditions, in the field. The effect of CO_2_ on the growth of diatoms is quite complex and, probably, depends on the particular physiology of each species. It has been shown that species can use different carbon sources, with some utilizing CO_2_ as main carbon source, whereas others mostly drawing carbon from HCO_3_^-^ [35]. Elevated CO_2_ concentration did not cause significant differences in growth in diatoms such as *Asterionella glacialis*, *Thalassiosira punctigera*, *Coscinodiscus wailesii*, *Phaeodactylum tricornutum* [36–38]. In the case of *Chaetoceros gracilis*, the maximum number of cells was obtained at a carbon dioxide concentration of 385 μatm and a lower cell number was obtained at lower (control and 280 μatm) and higher levels of carbon dioxide (1,050 μatm) [35]. In other species, such as *Thalassiosira weissflogii*, algal density decreased with the decreasing pH [39] probably because, when the acidity is lower than a certain concentration, it will impair algal physiological functioning [40]. In contrast, previous studies [41] demonstrated an advantage of larger planktonic diatom species, more than 40 μm in diameter, over smaller-sized ones with an enhanced growth rate under elevated pCO_2_ due to a combination of increased diffusion rates, a lowering of metabolic costs and a lower susceptibility to photo-inactivation of PSII.

*Cocconeis* spp., particularly abundant in the field at both normal and acidified areas of Castello Aragonese (Ischia, Naples, Italy), are a food source of *H*. *inermis* as demonstrated by the abundance of their thecae in its gut contents [42], especially in spring. Diatoms have been demonstrated to influence the ecology and the life cycle of other crustaceans [43–45] but in this species, according to co-evolutionary processes (triggering, in the shrimp, the development of beta females due to apoptotic disruption of the male gonadic buds;[42]), the toxic effect of diatoms are translated into their role as spring signals to set the reproductive cycle. Although it is known that acidification produces a change in the set of secondary metabolites produced by diatoms [22], here we demonstrated that the plant-animal relationship between *C*. *scutellum parva* and *H*. *inermis* is deeply affected by O.A. Although *H*. *inermis* is a polytrophic species [42], it strongly depends on *Cocconeis sp*. to keep the size of natural stocks constant. The development of beta females has been demonstrated to be a crucial factor in maintaining a constant sex ratio in this species, allowing for a fall large reproductive burst [6].

To obtain correct bioassays responses, the quality of shrimp larvae is of primary importance. For this reason, it is important to follow survival and growth, in larval and post-larval cultures of *H*. *inermis*, to evaluate their specific stress levels [46,47]. The reduction of stress factors, in studies on physiology of model organisms, should be taken into account to avoid bias in the reaching of actual sex ratios. Indeed, it was demonstrated that stress may influence sex ratios in protandric decapods [33,48,49]. Number of larvae produced by each female (81.8 ±19.3), low larval mortality and the duration of the larval period were in agreement to the health status of cultures in previous studies on *H*. *inermis* [7,8,50–52] and they may indicate absence of stress in cultured shrimps. A further demonstration of the low level of stress reached in larval and post-larval cultures was given by the low mortality, the size reached by most shrimps at the end of the feeding experiments and the low female/mature ratio observed in negative controls, as compared to previous studies [7,8,33].

Our bioassays confirmed the activity of *C*. *scutellum parva* compounds targeted the androgenic gland of *H*. *inermis* post-larvae [6–8,33,42]. It is worth observing that *Cocconeis scutellum parva* cultured in normal conditions (pH 8.2) was the most effective diatom with a 68.5 (±2.8) % females. mature individuals^-1^. On the contrary, *Cocconeis scutellum parva* cultured in acidified conditions produced the lowest female/matures ratio, with no significant differences in comparison to negative controls.

In order to culture highly adhesive benthic diatoms, the use of photobioreactors is convenient due to low operational time and perfect repeatability of procedures and it permits an optimization of space in thermostatic chambers [15] avoiding time consuming procedures of cultures in Petri dishes [53]. Various custom-made photobioreactors were designed to mass culture planktonic [54–56] and low adhesive benthic microalgae [57–59] but a few of them are specific for high adhesive benthic diatoms and, at the same time, are capable of manipulating pH [60]. The photobioreactor here described was proven to be effective in manipulating the pH in microalgal cultures and, in addition, they demonstrated to be capable of culture slow-growing, highly adhesive, benthic diatoms in axenic conditions.

It has been demonstrated that *Posidonia oceanica* meadows growing in acidified conditions show altered epiphyte and vagile fauna communities [61], with a strong reduction in organisms bearing aragonite skeletons. The seasonal correlation of the life cycle of *H*. *inermis* with the patterns of abundance of epiphytic *Cocconeis sp*. in *Posidonia oceanica* meadows [6] will be modified by climate change. In the future, *H*. *inermis* is forecasted to still be able to find *Cocconeis* spp. in the field, as the dominant epiphyte in acidified meadows [62] but the plant-animal co-evolutionary relationship will be probably lost, due to changes in the secondary metabolites produced by the microalga. For this reason, *Hippolyte inermis* could miss, in future, the possibility to obtain crucial infochemicals (e.g., the still unidentified apoptogenic compound) fundamental to triggering the apoptosis of cells in the androgenic gland of these shrimps facilitating its sex reversal. In an acidified environment, the abundance of *Cocconeis* spp. increase but this correspond to a lack of some key metabolites which impacts *H*. *inermis* life cycle, modifying its peculiar sex ratio.

The present study demonstrates that, besides basic processes directly influencing the life and the production of marine organisms, more complex mechanisms will determine the future of marine associations in acidified oceans.

## Supporting information

S1 Table Statistical analyses on the bioassay on *H*. *inermis*. A) ONE-way ANOVA compared the percentage of female/mature individuals among treatments. B) Tukey's multiple comparisons test among treatments.
(XLSX)

S2 Table Minimal data sets. (XLSX)
